# Pheochromocytomas and Paragangliomas: From Genetic Diversity to Targeted Therapies

**DOI:** 10.3390/cancers11040436

**Published:** 2019-03-28

**Authors:** Ying Pang, Yang Liu, Karel Pacak, Chunzhang Yang

**Affiliations:** 1 Section on Medical Neuroendocrinology, Eunice Kennedy Shriver National Institute of Child Health and Human Development, National Institutes of Health, Bethesda, MD 20892, USA; ying.pang@nih.gov (Y.P.); karel@mail.nih.gov (K.P.); 2 Neuro-Oncology Branch, Center for Cancer Research, National Cancer Institute, Bethesda, MD 20892, USA; yang.liu5@nih.gov

**Keywords:** pheochromocytoma, paraganglioma, neuroendocrine tumor, targeted therapy, therapy resistance

## Abstract

Pheochromocytoma and paraganglioma (PCPGs) are rare neuroendocrine tumors that arise from the chromaffin tissue of adrenal medulla and sympathetic ganglia. Although metastatic PCPGs account for only 10% of clinical cases, morbidity and mortality are high because of the uncontrollable mass effect and catecholamine level generated by these tumors. Despite our expanding knowledge of PCPG genetics, the clinical options to effectively suppress PCPG progression remain limited. Several recent translational studies revealed that PCPGs with different molecular subtypes exhibit distinctive oncogenic pathways and spectrum of therapy resistance. This suggests that therapeutics can be adjusted based on the signature molecular and metabolic pathways of PCPGs. In this review, we summarized the latest findings on PCPG genetics, novel therapeutic targets, and perspectives for future personalized medicine.

## 1. Introduction

Pheochromocytomas and paragangliomas (PGPCs) are catecholamine-producing tumors that arise from adrenal medulla, or from extra-adrenal ganglial sympathetic/parasympathetic chains (of chromaffin or non-chromaffin origin), respectively. Tumor-associated secretion of catecholamine causes symptoms of hyperactivity in the sympathetic nervous system including paroxysmal hypertension, headache and diaphoresis. PCPGs result from genetic abnormalities, mostly disruption/mutation in single disease-related genes [1]. Approximately 30–35% of patients with PCPG carry germline mutations in over 20 susceptible genes [2]. In pediatric patients, or in patients who developed the origin tumor in their childhood, approximately 69–87.5% of cases carry germline mutations [3]. Germline mutations may lead to clinical syndromes with symptoms that affect multiple organs, such as von Hippel-Lindau disease, multiple endocrine neoplasia type 2 syndrome, and neurofibromatosis type 1 [4]. On the other hand, somatic mutations in key oncogenic pathways, such as *SDHx, VHL, HIF2A, H-RAS, NF1, RET*, or *MAX,* predispose PCPG formation [5].

Despite our expanding understanding of PCPG genetics and transcriptomics, therapies against this malignancy, especially those against PCPG metastatic lesions, are limited. In addition to surgical resection and radiation therapy, combination chemotherapy that includes cyclophosphamide–vincristine–dacarbazine (CVD) is recommended for advanced PCPG. However, retrospective studies showed that CVD-based treatment provides limited benefit to patient quality of life and overall survival [6]. There is an urgent need to decipher the molecular signature of PCPG for optimized therapeutic regimens, which may result in improved selectivity and efficacy of treatment. In this review, we summarized the latest reports on PCPG genetics, clinical findings and management, and emerging targeted therapies against PCPG subtypes.

## 2. Genetics of PCPGs

**Figure 1:**
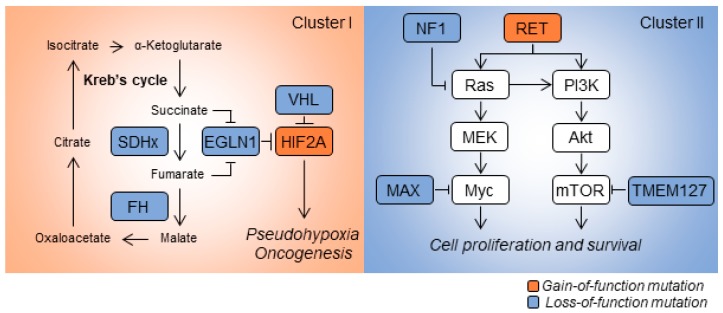
Schematic illustrations of cancer-associated mutations in pheochromocytomas and paragangliomas (PCPGs). Cluster I PCPGs exhibit dysfunction in the Krebs cycle and hypoxia sensing pathways. Loss-of-function mutations in *SDHx*, *FH*, *EGLN1* or *VHL* are commonly identified in this disease cluster. *HIF2A* mutations that activate hypoxia signaling are also found in Cluster I disease. Cluster II PCPGs exhibit abnormal kinase activity. This is caused by mutations of major regulators in the feedback loop, such as *NF1*, *MAX* and *TMEM127*. Gain-of-function mutations in *RET* prompt cellular proliferation and survival by initiating kinase pathways such as Ras/MEK and PI3K/Akt.

Transcriptomic analysis of patient-derived specimens revealed distinctive gene-expression signatures among histologically similar PCPGs. Based on mRNA-expression signatures, PCPGs can be divided into two main categories: Cluster I and Cluster II diseases (Figure 1). Cluster I disease exhibits metabolic reprogramming and pseudo hypoxic signaling commonly linked to mutations in oxygen-sensing genes or those encoding key enzymes in the Krebs cycle such as *VHL, SDHx, HIF2A, EGLN1/2* and *FH*. Cluster I disease is further stratified into respective subgroups based on differentially-expressed genes. PCPGs showing mutation of *SDHx* and *VHL* are sub-characterized into Cluster IA and Cluster IB, respectively [5]. In contrast, Cluster II PCPGs are commonly related to genetic mutations affecting kinase signaling, gene translation, protein synthesis and neural differentiation; the genes showing mutations include *NF1, RET, KIF1Bβ*, *TMEM127* and *MAX*. Cluster II disease is further categorized into Cluster 2A (in which patient show mutations in *RET, NF1,* and *TMEM127* ), Cluster 2B (sporadic tumors) and Cluster 2C (patients with mutations in 3.7% *VHL* and 11.1% *RET,* and sporadic tumors) [5]. Recent findings show that mutations in the Wnt/Hedgehog pathway are involved in a new molecular subtype of PCPGs [7]. Fishbein et al. discovered that the in-frame RNA fusion transcripts of the *UBTF-MAML3* gene and somatic *CSDE1* mutation may drive activation of the Wnt and Hedgehog pathways, and trigger PCPG oncogenesis [8]. In addition to assessing mutations in coding sequences, analysis of somatic copy-number alterations and miRNA profiling are increasingly used to determine sub-clusters in PCPGs [9].

### 2.1. SDHx

Germline mutations in *SDHx* are attributed to approximately half of hereditary PCPGs and are detected in 15% of total patients [10]. Germline mutations in *SDHx* are commonly accompanied by the loss of heterozygosity on the other healthy allele, which leads to substantial loss of SDH catalytic activity [11]. Familial PCPGs, caused by *SDHx* germline mutations, usually show earlier onsets and more severe clinical presentations (including bilateral or multiple tumors) compared with those observed in sporadic cases [12]. In 2000, *SDHC* and *SDHD* were first identified as susceptibility genes for hereditary PCPGs [13,14]. *SDHC* mutations account for 6% of PCPGs, and patients usually present head and neck paragangliomas (HNPGL), while PHEO and PGL occur far less frequently [15]. *SDHD*-mutant PCPGs typically show multiple HNPGL, but PGL and PHEO in other locales have also been described; less than 5% of patients with *SDHD* mutations develop metastatic lesions [15]. Overall, the penetrance of *SDHD*-mutant PCPGs is approximately 71% at age 60 and increases to 90% in the following 10 years [16]. Germline mutations of *SDHD* exhibit ‘parent-of-origin’ expression phenotype, with tumor onset only when mutations are inherited from the paternal DNA [17,18]. This phenomenon has also been described in other PCPG predisposition genes such as *SDHAF2* and *MAX* [19,20,21]. In 2001, mutations in *SDHB* were also discovered in patients with familial PCPG [22]. *SDHB*-mutant tumors can occur at adrenal, extra-adrenal and pelvic locations, but mainly develop in the abdomen. Several studies demonstrated that compared with other molecular subtypes, *SDHB*-mutant PCPGs are associated with increased incidence of early onset (25–30 years old), increased metastatic risk and poor prognosis [23]. In 2009, *SDHAF2*, also known as *SDH5*, was identified as the driver gene for HNPGL without PHEO, which occurs via compromised flavination of the SDH complex [19]. In patients with familial PCPGs who carry *SDHAF2* germline mutations, 91% present with more than one HNPGL, and no metastatic tumors have been reported [24]. Mutations in *SDHA* have not been identified as a cancer susceptibility gene in PCPG until recently [20]. Approximately 3% of patients with sporadic PCPG carry *SDHA* germline mutations [25]. Somatic mutations in *SDHx* are rare and occur in approximately 1% of patients with PCPG [5].

*SDHx* genes encode succinate dehydrogenase (SDH), also known as mitochondrial complex II. SDH consists of four subunits: SDHA, SDHB, SDHC and SDHD. SDHA is a flavoprotein that contains a flavin adenine dinucleotide (FAD) cofactor. SDHB contains three iron-sulfur clusters, which assist electron transfer via the SDH complex. SDHC and SDHD subunits anchor the entire SDH complex to the inner mitochondrial membrane. Mechanistically, SDHA converts succinate into fumarate, which converts FAD to FADH_2_. The electrons from FADH_2_ are then transferred via iron-sulfur clusters in SDHB, eventually forming the ubiquinone pool via SDHC/D subunits. SDH complex plays key roles in energy metabolism by participating in both the Krebs cycle and electron transport chain. Deleterious mutations in *SDHx* lead to deficiencies in energy metabolism and accumulation of succinate, which promotes susceptibility to PCPGs, renal cell carcinoma and mitochondrial encephalopathy. Studies using in vivo and in vitro models have shown that loss of succinate dehydrogenase activity results in: (i) abnormal activation of hypoxia-signaling pathway in the presence of oxygen (pseudohypoxia) and angiogenesis [26]; (ii) increased production of reactive oxygen species (ROS) [27]; and (iii) impeded repair and hypermethylation of DNA [28]. The distinctive signatures in tumor biology have supplied valuable clues for developing future molecular-targeted therapeutics against *SDHx*-mutant PCPGs.

### 2.2. Von Hippel-Lindau (VHL)

Germline mutations in the *VHL* gene cause the von Hippel-Lindau syndrome (VHL disease). VHL disease is an autosomal dominant disorder associated with retinal, cerebellar, brainstem and spinal hemangioblastoma, as well as with neuroendocrine tumors, renal cell carcinoma (RCC) and multiple pancreatic cysts [29]. PHEO is present in approximately 7–20% of patients with VHL, who are then diagnosed with VHL syndrome type 2; patients diagnosed with type 1 VHL do not present with PHEO [30]. PHEO usually occurs as bilateral or multifocal tumors in the second decade of life in patients with VHL. Although *VHL* mutations lead to early onset of symptoms, they rarely develop into metastatic disease. In addition to the VHL syndrome, Chuvash polycythemia is a type of inherited hematopoetic disease caused by a specific germline *VHL* mutation (p.R200W). The mutation leads to activation of the hypoxia inducible factors (HIF) signaling pathway under normal oxygen level and increased concentration of erythropoietin, causing overproduction of red blood cells [31]. Germline *VHL* pathogenic mutations are also reported in patients with PHEO and polycythemia, causing by stabilized HIF-2α and elevated production of erythropoietin [32].

Approximately 14% of sporadic PCPGs are found in patients carrying somatic *VHL* mutations, and this is consistently accompanied by the loss of the 3p chromosome [5]. Our previous study has shown that somatic *VHL* gene mutations are also involved in tumorigenesis in hereditary MEN 2A-associatd PHEO [33]. Somatic *VHL* mutations play roles in HNPGL by stimulating the HIF-1α/miR-210 pathway [34]. Although the relationship between somatic *VHL* mutations and prognosis is unclear, different *VHL* variants may contribute to the differential clinical phenotype and prognosis. In our recent study, we established a *VHL* knockout mouse model and found that retinal hemangioblastomas are derived from the hemangioblast cell lineage [35].

*VHL* is a tumor-suppressor gene that is located on chromosome 3p25.3 and encodes the pVHL protein. The pVHL protein functions as an E3 ligase that ubiquitinates its client proteins. For example, pVHL recognizes the hydroxylated HIF-α oxygen-sensing domains (ODD) domain and recruits other components of the E3 ligase complex such as Elongin B, Elongin C, RBx 1 and Cul2. The VHL-Elongin B/C (VBC) complex processes HIF-α for ubiquitination and subsequent proteasomal degradation. Under hypoxic conditions, VHL recognition of HIF-α is compromised due to reduced ODD hydroxylation. HIF-α is then stabilized and initiates transcription of hypoxia-related genes. Pathogenic *VHL* mutations lead to compromised VBC activity and abnormal oxygen sensing. Consequent transcription of hypoxia-related genes, such as *EPO* and *VEGFA*, serves as oncogenic factors for *VHL*-related symptoms such as hemangioblastomas and PHEO. Moreover, mutations in *VHL* may disrupt the binding of Elongin C and p53, leading to deregulation of cellular apoptosis and consequent tumorigenesis [36].

### 2.3. HIF2A

Hypoxia inducible factors (HIFs), transcriptional factors that govern cellular responses to low oxygen, were first described by Semenza in 1995 [37]. HIFs are composed of α and β subunits. The α subunits are nuclear factors that are sensitive to the oxygen level in the microenvironment, whereas β subunits are constitutively expressed and serve as cofactors for HIF-α. Under normoxia, the ODD in HIF-α are rapidly hydroxylated by prolyl hydroxylase, which alters the conformation of HIF-α. Hydroxylated HIF-α is recognized by the VBC complex and is rapidly degraded via the ubiquitin proteasome pathway [38]. Under hypoxic or pseudohypoxic conditions, the function of prolyl hydroxylase is compromised, leading to stabilization and accumulation of HIF-α. HIF-α is then translocated into the nucleus as a heterodimer HIF-β, initiating transcriptional activation of hypoxia-related genes involved in biological reactions such as angiogenesis, glycolysis and erythropoiesis.

Overexpression of HIF-1/2α is frequently identified in most human cancers, and activation of tumorigenesis and angiogenesis [39]. Low oxygen concentration activates the hypoxia-signaling pathway in tumors, especially in regions with minimal oxygen penetration. On the other hand, the hypoxia pathway can also be activated under normoxia due to genetic abnormalities in key regulatory genes of the oxygen-sensing pathway. Elevated expression of HIF-1α is associated with poor outcomes in multiple human cancers such as those of head and neck, breast and colorectal cancers [40]. HIF-2α overexpression is associated with higher metastatic potential and with metastases-presenting tumors such as melanoma and glioma [41]. HIF-2α overexpression may be preferentially linked with metastatic progression and poor prognosis in patients [41].

Mutations in *HIF2A* have been identified in human diseases such as polycythemia, PCPG and somatostatinoma [42,43]. *HIF2A* mutations present as somatic mutations or somatic mosaicism, affecting multiple lineages of somatic cells [44]. *HIF2A* mutations are mainly located on exon 12, resulting in amino-acid substitutions in the ODD domain of HIF-2α. Alterations in peptide sequences lead to compromised prolyl hydroxylation, VBC recognition and transcription of hypoxia-related genes. Accordingly, *HIF2A*-mutated PHEO/PGLs show increased expression of hypoxia-related genes such as *EPO*, *EDN1* and *VEGFA*, which may be linked to polycythemia and oncogenesis [43].

### 2.4. Neurofibromin 1 (NF1)

The NF1 syndrome, also known as von Recklinghausen disease, is caused by germline mutations in *NF1*. Mutations in *NF1* are involved in numerous types of tumors such as desmoplastic melanoma, glioblastomas, neuroblastomas, PCPGs, gastrointestinal tumors, ovarian tumors and urinary tract transitional cell carcinoma [45]. Approximately 0.1–6% of patients with NF1 present with PHEO [46]. Patients with NF1 usually develop PHEO after their third decade of life. Approximately 80% of these patients present solitary adrenal tumors, and 10% present with bilateral adrenal tumors. Additionally, over 10% of patients with NF1 and PHEO develop metastatic tumors, and most of these metastatic tumors are distant from the primary location [47]. A common feature observed in patients with *NF1*-related PCPGs is a significantly up-regulated level of catecholamine in plasma and urine [48].

Somatic mutations in *NF1* occur in 20–25% of patients with PCPGs. *NF1* is the most frequently occurring susceptibility gene in all sporadic PCPGs. An integrative genomic study has shown that 26% of sporadically occurring tumors show loss of one allele in *NF1*. Additionally, 91% of tumors in patients with *NF1*-related PCPGs show somatic truncating mutations on the other wild-type allele [48]. However, a genetic-mapping study has shown that only 20% of patients with *NF1*-related PCPGs show deletion of the other allele [5], indicating that other molecular pathways may be involved in NF1-mediated oncogenesis.

*NF1* is a tumor-suppressor gene located on chromosome 17q11.2. The *NF1* gene spans approximately 300 kb in genomic DNA, contains 58 coding exons and encodes 2818 amino acids. Currently, genetic detection and characterization of *NF1* mutations in patients is challenging because of the large size of the *NF1* gene, presence of multiple pseudogenes, and a wide spectrum of mutations without obvious hotspots. *NF1* encodes neurofibromin, a GTPase-activating protein (GAP) that negatively regulates the Ras/MAPK pathway. The 20 to 27 exons of *NF1* encode a GAP-associated domain, which hydrolyses Ras-GTP to its inactive GDP-bound form, thereby deactivating the Ras signaling pathway. Loss-of-function mutations in *NF1* lead to uncontrollable activation of kinase and tumorigenesis. Several genetically-engineered *NF1* mouse models have shown pigmentary lesions, skeletal abnormalities, and tumors.

### 2.5. RET

Germline mutations in *RET* are linked with multiple endocrine neoplasia type 2 (MEN2). MEN2 is a rare autosomal dominant syndrome that is classified into MEN2A (Sipple syndrome), MEN2B (Gorlin syndrome) and familial medullary thyroid carcinoma (FMTC). Patients with MEN2 have a nearly 100% risk for developing medullary thyroid carcinoma (MTC) and 57% risk for developing PHEO [49]. Additionally, patients with MEN2A can also develop primary hyperparathyroidism, while those with MEN2B can develop Marfanoid habitus, mucosal neuromas and ganglioneuromatosis. Although mutations in *RET* have been detected on all exons, 95% of patients with MEN2A carry *RET* mutations on exon 10 (codons 609, 611, 618 and 620) or exon 11 (codon 634). Similarly, most mutations in patients with MEN2B occur on exon 16 (codon 918) [50]. The most common *RET* mutations in PHEO-related syndrome usually occur on exon 10, 11, 13 and 16. However, penetrance and age of onset are not necessarily associated with types of *RET* mutations [51]. Carriers of codon 634 germline mutations present with much younger mean age of onset, and have a higher risk of developing PHEO, than do carriers of other mutations. In patients with MEN2, most PCPG-related PHEOs occur on the adrenal glands, and more than half of these are bilateral; parasympathetic head and neck PGLs have been found, but are very rare [52]. These patients rarely develop metastatic PCPGs, and mean age of onset is approximately 36 years old [53].

The *RET* proto-oncogene is located on chromosome 10q11.2 and contains 21 exons. *RET* encodes transmembrane receptor tyrosine kinase (RTK), which binds to growth factors such as glial derived neurotrophic factor (GDNF). The RET protein contains an extracellular portion, a single transmembrane domain and an intracellular portion. There are 12 autophosphorylation sites on the intracellular portion, and phosphorylated tyrosine may be the docking site for multiple intracellular-signaling pathway proteins, including those involved in cell growth and differentiation [54]. Genetic alterations in *RET* include gain-of-function mutations, which lead to constitutive RTK activation and tumorigenesis such as those observed in patients with MEN2A.

### 2.6. MAX

Germline mutations in *MAX* were first implicated in susceptibility to hereditary PHEO in a whole-exome sequencing study. Loss-of-function mutations in *MAX* are also a risk for metastatic PHEO [21]. Most of the *MAX* mutations occur on the highly-conserved basic helix-loop-helix leucine-zipper (bHLHZ) domain. Loss of heterozygosity on the wild-type allele is also detected in the tumors of patients with germline missense mutations in *MAX*. Although metastatic PHEOs are rare, except in patients carrying *SDHB* mutations, Mendez found that approximately 37% of patients with *MAX* mutations present with metastases at diagnosis [21]; this suggests *MAX* mutations may be risk factors for metastatic disease. Somatic *MAX* mutations are detected in patients with sporadic PCPGs at an incidence of 1.65% [55]. Tumors with *MAX* mutations show substantial upregulation of normetanephrine expression, with almost normal or slighted increased levels of metanephrine.

The *MAX* gene is located on chromosome 14q23, which encodes the transcriptional regulator MAX. MAX belongs to the family of bHLHZ transcriptional factors. It can form heterodimers with MYC or MAX dimerization protein 1 (MXD1), which controls the transcription of numerous downstream genes that regulate cellular proliferation, differentiation and apoptosis [56]. The highly-conserved bHLHZ domain of MAX is vital for the protein-DNA and protein-protein interactions. Furthermore, casein kinase II phosphorylation sites on MAX modulate DNA-binding kinetics of MAX-MAX or Myc-MAX dimerization [57]. Therefore, alteration in MAX, especially mutations on the bHLHZ domain and casein kinase II phosphorylation sites, can induce the dysfunction of the MYC/MAX/MXD1 axis and consequent tumorigenesis.

### 2.7. Harvey Rat Sarcoma Viral Oncogene Homologue (HRAS)

The first somatic mutation in *HRAS* in a patient with pheochromocytoma was reported by Yoshimoto et al. in 1992 [58]. Missense gain-of-function mutations in *HRAS* have been detected in various types of human tumors; the hotspots for *HRAS* mutations are G13R and Q61K [1]. Until now, *HRAS* somatic mutations were found in approximately 5% of sporadic patients with PCPGs and present as mostly benign tumors [1]. No germline HRAS mutation has been discovered in patients with PCPGs thus far. The other two proteins in the RAS family, NRAS and KRAS, have never been described as susceptibility factors for PCPGs.

*HRAS* is located on the chromosome 11p15.5. *HRAS* encodes GTPase HRas, also known as transforming protein p21. HRas is activated via binding to GTP. The activity of HRas can be inactivated by GTP hydrolysis to GDP [9]. Activation of the HRas signaling pathway stimulates downstream pathways such as Ras/Raf/Erk and PI3K/Akt/mTOR, which are vital for cellular proliferation and oncogenic transformation.

## 3. Current Therapies and Limitations

The goal of anti-PCPG therapies is to effectively control tumor growth and other disease-related symptoms. Alpha-blockers, calcium channel blockers, or β-blockers are the first line treatment to control hypertension and prevent hypertensive crisis. When β blockers are used without prior alpha blockade, there is a theoretical risk of hypertensive crisis due to alpha adrenergic receptor mediated vasoconstriction without the opposition of the β2-adrenergic receptor mediated vasodilation. For benign and locally invasive PCPGs, surgical intervention, including minimal invasion endoscopic surgery, is considered standard therapy. Laparoscopic surgery can be used for patients with bilateral and extra-adrenal PCPGs, with laparotomy showing similar outcomes. For multifocal and metastatic cases, and for tumors larger than 7 to 8 cm, surgical procedures are usually preferable for ensuring complete removal of all suspected tumors. When surgery is not applicable, radio-and/or chemotherapies are considered alternative approaches. For the metaiodobenzylguanidine (MIBG) scintigraphy-positive patients, ^131^iodine-meta-iodobenzylguanidine (^131^I-MIBG) therapy is considered a priority. MIBG positive patients with metastatic PCPG have been demonstrated to benefit from ^131^I-MIBG-based treatment, showing symptomatic and hormonal responses [59]. However, dose-dependent side effects of this therapy, such as severe thrombocytopenia, hypothyroidism and neutropenia, are also observed [60]. Most importantly, ^131^I-MIBG-based treatment is less likely to achieve complete response. In a study that included 243 patients, 3% of patients showed complete response, while 27% and 52% of patients showed partial response and stable disease, respectively [61].

Overexpression of somatostatin receptors in PCPGs promotes application of radiolabeled somatostatin agonists, for imaging and treatment of the PCPGs patients. ^123^I-Tyr-octreotide and ^111^In-pentetreotide were first introduced as the radiolabeled somatostatin agonists. However, the ^90^Y and ^177^Lu peptide-labelled somatostatin radionuclides were recommended by European centers to replace the old ones, due to higher uptake ratio and less side effects [62,63]. Besides, the ^90^Y is more effective on larger tumors due to higher energy β emission, while the ^177^Lu is favorable for smaller tumors. Less side effects were also found in ^177^Lu compared to ^90^Y, especially in the aspect of renal toxicity [64]. A successful phase III clinical trial NETTER-1 regarding the ^177^Lu-DOTATATE showed to prolong the median progression-free survival to 40 months in mid-gut neuroendocrine tumors, compared to a long-acting somatostatin analogue, octreotide-LAR (median progression-free survival: 8.4 months) [65]. For inoperable PCPGs patients, the ^177^Lu-DOTATATE is under a phase II clinical trial to evaluate the safety, tolerability and overall survival (NCT03206060). However, radiolabeled somatostatin agonists are only applied for somatostatin receptor positive patients and side effects still need further evaluation.

Chemotherapy is another valuable treatment modality for controlling tumor growth in patients with metastatic PCPGs. Most traditional chemotherapy regimens, such as those using cyclophosphamide, vincristine and dacarbazine (CVD), have been used to treat patients with PCPGs over the past 30 years. Although clinical studies have shown that 33–57% of patients with PCPGs respond to CVD or similar regimens, a 22 year-long follow-up study found there were no significant differences in patient survival between CVD responders and CVD non-responders. Overall, the present options of chemotherapy do not provide survival benefits for advanced PCPG, and their value remains limited [6].

## 4. Targeted Molecular Therapies

Current knowledge of signatures involved in the molecular signaling, metabolism and resistance mechanisms of PCPGs suggests that therapeutic regimens can be optimized to each molecular subtype. Profiling of gene expression and methylation can serve as a powerful tool for characterizing disease clusters and for guiding targeted therapy for improved selectivity and efficacy. In the following sections, we introduce the latest advances in targeted therapeutics against PHEO/PGL.

### 4.1. Antiangiogenic Therapies

Antiangiogenic therapies have been proposed for targeting pseudohypoxic and angiogenic phenotypes in Cluster I PCPGs, which are commonly accompanied by mutations in *SDH* or *VHL* [66]. Humanized VEGF-A monoclonal antibodies (such as bevacizumab) and tyrosine kinase inhibitors (such as sunitinib and sorafenib) are used in current antiangiogenic therapies. These regimens are approved by the FDA for the treatment of patients with advanced renal cell carcinoma, which includes patients with mutations in *SDHB* [67,68]. Interestingly, several case studies on sunitinib have shown partial response or stable disease in patients with Cluster I PCPGs. This indicates that patients with Cluster I PCPGs may show improved responses to antiangiogenic therapies [69,70,71,72,73,74]. Several ongoing clinical trials are aiming to further validate the efficacy of sunitinib-based therapy in patients with progressive PCPGs. For example, a randomized double-blind phase II clinical trial, called the FIRSTMAPPP (First Randomized STudy in MAlignant Progressive Pheochromocytomas and Paragangliomas) study (NCT01371201), is currently conducting recruitment to evaluate the efficacy of sunitinib vs placebo in patients with progressive malignant PCPGs. A single arm, nonrandomized phase II study (NCT00843037) aims to evaluate the response and toxicity profile of sunitinib in a cohort of 25 patients with malignant PCPGs. Another tyrosine kinase inhibitor, Axitinib (AG-013736), is currently under evaluation in a phase II nonrandomized clinical trial including 14 patients with PCPGs (NCT01967576). Moreover, a phase II clinical trial is ongoing to determine the efficacy of Lenvatinib, a multiple kinase inhibitor against VEGFR1, VEGFR2 and VEGFR3 in patients with metastatic or advanced PCPGs (NCT03008369).

### 4.2. Hypoxia-Inducible Factor (HIF) Inhibitors

The abnormal activation of hypoxia signaling is a hallmark of Cluster I PCPGs. HIF inhibitors may potentially be used in therapy against Cluster I PCPGs. HIF inhibitors, such as PX-12 and PX-478, have been studied in various tumor xenograft models [75,76]. Recently, PT2339 and PT2385, two selective HIF-2α antagonists, were developed and evaluated for their anti-tumor effects. PT2399 showed a stronger suppression effect than that of sunitinib in cell lines derived from *VHL*-mutated clear cell renal cell carcinomas (ccRCCs) [77]. An ongoing phase I clinical trial (NCT02293980), designed to evaluate the efficacy of PT2385, indicated that complete response, partial response and stable disease were achieved in 2%, 12% and 52% of patients with ccRCCs [78]. A phase II clinical trial (NCT03108066) is currently ongoing to evaluate the use of PT2385 in patients with *VHL*-associated ccRCCs. These compounds have not been evaluated in patients with PCPGs; however, the tumor-suppressing effects of these compounds on HIF-driven solid tumors are promising, suggesting that HIF-2α inhibitors can be used to treat patients with Cluster I PCPGs in the future. Recently, anthracyclines (daunorubicin, doxorubicin, epirubicin and idarubicin) have been reported to suppress cell growth of metastatic PCPGs by inhibiting both HIF-1 and 2α, indicating a new therapeutic option for patients with metastatic PCPGs, especially those with alterations in HIF pathways [79].

### 4.3. mTOR Inhibitors

Hyperactivation of kinase activity is commonly detected in the Ras/Raf/Erk or PI3K/Akt/mTOR pathways of patients with Cluster II PCPGs and mutations in *RET*, *NF1*, *TMEM127* and *MAX,* [46,55,80,81,82]. Inhibitors of pro-survival kinase signaling have been proposed for targeted therapeutics. For example, treatment with mTORC1 inhibitor everolimus (RAD001) has been evaluated in patients with progressive PHEO. However, this therapy showed unfavorable results, with disease progression in all four recruited patients [83]. In another phase II study (NCT01152827), five out of seven patients with PCPGs achieved stable disease [84]. In 2013, a selective ATP-competitive dual mTORC1/2 small molecule inhibitor was evaluated in a mouse model of sporadic PHEO, and PHEO associated with *VHL* or *SDHB* mutations. The results showed promising therapeutic effects of AZD8055, indicated by decreased tumor size and metastatic burden in athymic nude mice [85]. Moreover, combining AZD8055 with an Erk inhibitor AEZS-131 may prevent the compensatory feedback loop and overcome resistance [86].

### 4.4. DNA Demethylation

Mutations in *SDHx* result in accumulation of succinate, an oncometabolite that inhibits 2-oxoglutarate (2-OG)-dependent dioxygenases, resulting in a global DNA and histone hypermethylation phenotype [87,88]. Demethylating agents may rectify the hypermethylation phenotype in *SDH*- or *FH*-mutated PCPGs. For example, DNA-demethylating agent decitabine suppresses cellular proliferation and metastasis in *SDHB*-knockout chromaffin cells [87]. SGI-110, a DNA methyltransferase inhibitor, is currently under investigation in a phase II non-randomized trial (NCT03165721) for treatment of patients with PCPGs associated with SDH deficiency. Further preclinical studies are needed to assess the safety profile and therapeutic efficacy of these compounds before proceeding to clinical trials.

### 4.5. DNA-Alkylating Agents

Temozolomide (TMZ) is an FDA-approved DNA-alkylating agent used for treatment of glioblastoma in combination with radiotherapy. TMZ generates DNA alkylation at O6-guanine, N7-guanine and N3-adenine, which causes base-pair mismatch and leads to the death of tumor cells. In some tumor cells, the expression of O6-methylguanine-DNA methyltransferase (MGMT) can directly remove the alkyl group from O6-guanine, resulting in resistance to TMZ. However, tumors with mutations in genes encoding Krebs-cycle enzymes, such as *IDH1/2* and *SDHx*, often show CpG island methylator phenotype (CIMP), which results in hypermethylation of the *MGMT* promoter and reduced expression of MGMT [87,89]. Loss of MGMT expression predisposes patients to a better therapeutic response to TMZ because of reduced methyltransferase activity. Several studies have shown the remarkable sensitivity of *IDH1/2*-mutant glioblastoma to TMZ [90,91]. Similarly, TMZ exerts strong therapeutic effect on metastatic neuroendocrine carcinoma, especially that with mutations in *SDHB* [92]. A phase II clinical trial (NCT00165230) is currently evaluating the efficacy of TMZ combined with thalidomide in therapy against neuroendocrine tumors. Additionally, one out of three patients with PCPGs shows response to radiotherapy [93]. Clinical trials with larger patient cohorts are needed to further evaluate the efficacy of TMZ in patients with PCPGs.

### 4.6. PARP Inhibitors

Mutations in enzymes encoding Krebs-cycle enzymes, such as *SDHx*, are associated with hereditary PCPGs that are characterized by increased level of succinate. High level of succinate serves as an intrinsic inhibitor of homologous recombination (HR)-based DNA repair; this occurs via inhibition of the lysine demethylases KDM4A and KDM4B [94]. Moreover, SDH deficiency in Cluster I PCPGs is associated with alterations in NAD^+^/NADH metabolism and potentiation of the PARP-mediated DNA repair pathways [95]. These findings indicate that SDH-deficient tumor cells are highly sensitive to treatment with PARP inhibitors. Combinations of PARP inhibitors with other genotoxic agents may be a promising approach for treating patients with Cluster I PCPGs. Olaparib, an FDA-proved PARP inhibitor, markedly potentiates the therapeutic effect of TMZ in *SDHB*-mutant preclinical models; this occurs via induction of DNA lesions and inhibition of tumor growth in vitro and in vivo [94,95].

### 4.7. Histone Deacetylase Inhibitors

Histone deacetylase (HDAC) inhibitors were also reported to have anti-tumor effect in PCPGs. HDAC inhibitors have been shown to induce cell cycle arrest and apoptosis in PCPGs through activation of Notch1 signaling or inhibition of nuclear factor erythroid 2-related factor 2/heme oxygenase 1(Nrf2/HO-1) pathway [96,97,98,99]. Additionally, our previous study demonstrated that HDAC inhibitors improved the stability of SDHB protein, and therefore supported the function of mitochondrial complex II, which might limit disease progression of PCPGs with *SDHB* deficiency [100].

### 4.8. Immunotherapy

The pseudo-hypoxia phenotype may alter the immune system through inactivation of cytotoxic T-cell lymphocytes, activation of immune-suppressive monocytes and increased expression of the immune checkpoint protein programmed death-ligand 1 (PD-L1) and its receptor [101,102,103]. Thus, immunotherapy has been considered as a candidate therapeutic approach for Cluster I PCPGs. A study of 14 patients with progressive metastatic PCPGs treated with interferon alpha-2b resulted in 12 patients with disease stabilization and two with partial responses [104]. Two phase II clinical trials of checkpoint inhibitors (Nivolumab, ipilimumab and pembrolizumab) are currently ongoing in patients with rare tumors, including metastasis PCPGs (NCT02834013, NCT02721732).

### 4.9. Other Potential Therapies

Our previously study indicated that cells with high baseline level of reactive oxygen species (ROS), such as *IDH*-mutated glioma, dependency on antioxidative pathways are crucial to maintain ROS homeostasis. Blockade of antioxidative pathways showed promising therapeutic effects in *IDH*-mutated cancers [105]. Similarly, evidence has shown that the deficiency in *SDH* and accumulation of succinate may lead to elevated generation of ROS [27,106]. Our recent data indicated that *SDHB* deficient PCPG cells developed addiction to the Nrf2 antioxidative pathway and Nrf2 blockade might be a novel therapeutic approach to this type of PCPGs.

## 5. Future Directions

Despite our increased understanding of PCPG biology and advancements in translational medicine, the underlying pathogenetic mechanisms and molecular pathways of PCPG require further investigation. Cell-based and preclinical mouse models do not fully recapitulate the molecular subtypes of human cancers, posing a challenge in studies on PCPG. PCPG cell lines, derived from heterozygous *NF1*-knockout mouse (MTT and MPC cells) and representative rat pheochromocytoma (PC12 cells), are widely accepted and used in molecular biology studies; however, generating cell lines from patient-derived PCPGs remains challenging. Currently, only one progenitor cell line (hPheo1 cells) derived from a human pheochromocytoma tumor has been established successfully [107]. This illustrates an urgent need to develop patient-derived cell lines, especially those for modeling Cluster I PCPG in vitro.

With the rapid development of genomic sequencing platforms, large-scale sequencing projects have illustrated the genomics, methylomics and epigenenomic changes of PCPG. The concept of personalized medicine has been brought into vision to treating individuals based on their specific genetic and micro-environmental background. By altering specific signaling pathways, enzymes and receptors, targeted therapies can be optimized for each individual, with reduced side effects with respect to normal tissues. To this end, tumoral genetic and molecular profiles should be investigated in future clinical studies and trials. On the other hand, continuous investigation of molecular mechanisms involved in PCPG oncogenesis is highly important. Detailed understanding of PCPG genetics and key oncogenic pathways will lead to novel therapeutic targets. Overall, understanding the genetic background, developing effective molecular-targeted agents and optimizing the design of clinical trials will improve prognosis and survival in patients with PCPG.

## 6. Conclusions

In this review, we briefly summarized the latest knowledge of PCPG molecular subtypes and their implications to clinical management. PCPGs generate tumors with genetic alterations; therefore, detailed genetic analysis should be recommended for all patients with PCPGs to better characterize the potential therapeutic vulnerabilities in each case. Therapeutic regimens with long-term efficacy are needed to improve patient survival and quality of life. Development of patient-derived cell lines and disease-relevant preclinical animal models will generate novel therapeutic targets for future management of PCPGs.
